# Prognostic Factors and Survival of Chinese Patients with Cardiac Amyloidosis

**DOI:** 10.1155/2023/3070017

**Published:** 2023-05-16

**Authors:** Lu Zhang, Sulei Li, Bohan Liu, Jing Wang, Yang Mu, Xuan Zhou, Hongbin Liu

**Affiliations:** Department of Cardiology, The Second Medical Center & National Clinical Research Center for Geriatric Diseases, Chinese PLA General Hospital, Beijing, China

## Abstract

**Purpose:**

To identify the survival and prognostic factors for cardiac amyloidosis (CA) in Chinese patients.

**Methods:**

This was a prospective cohort study of 72 patients diagnosed with CA and admitted to the PLA General Hospital between November 2017 and April 2021. Demographic, clinical, laboratory, electrocardiographic, conventional ultrasound, endocardial LS during LV systole (LV ENDO LSsys), and myocardial strain data were recorded. Survival was assessed. All-cause mortality was the endpoint. Follow-up was censored on September 30, 2021.

**Results:**

The mean follow-up was 17.1 ± 12.9 months. Among the 72 patients, 39 died, 23 survived, and 10 were lost to follow-up. Mean survival for all patients was 24.7 ± 2.2 months. Mean survival was 32.7 ± 2.4 months among patients with NYHA class II, 26.6 ± 3.4 months for NYHA class III, and 5.8 ± 1.1 months for NYHA class IV. The multivariate Cox proportional hazard regression model showed that NYHA class (HR = 3.42, 95% CI: 1.36–8.65, *P* = 0.002), log-proBNP level (HR = 1.40, 95% CI: 1.17–5.83, *P* = 0.03), and ENDO LSsys of the LV basal level (HR = 1.25, 95% CI: 1.05–1.95, *P* = 0.004) were independent prognostic factors for CA.

**Conclusion:**

NYHA class, proBNP level, and ENDO LSsys of the LV basal level were independently associated with the survival of patients with CA.

## 1. Introduction

Cardiac amyloidosis (CA) is characterized by the deposition of amyloid plaques in the myocardial interstitium, leading to the disruption of normal cardiac structure and function and, consequently, to myocardial dysfunction and conduction abnormalities or systemic amyloidosis with cardiac involvement [1]. Cardiac involvement is the main cause of the high morbidity and mortality of systemic amyloidosis [2]. The median survival of patients with primary amyloidosis without cardiac involvement is about 4 years, but patients with cardiac involvement (AL-CA) and a significant increase in levels of N-terminal precursor of brain natriuretic peptide (NT-proBNP) or cardiac troponin T (TnT) have a poor prognosis, with a median survival of only 8 months [3, 4]. Recent international studies showed that hematological cardiac markers, conventional echocardiographic parameters, and Doppler parameters of cardiac ultrasound were associated with the prognosis of patients with AL-CA [5, 6], but studies on the prognosis of Chinese patients with CA and the factors that predict prognosis are limited.

Velocity vector imaging (VVI) is a quantitative two-dimensional grayscale echocardiographic modality that uses a more complex approach than the speckle-tracking method. It allows the 3-dimensional quantification of myocardial deformation in the different layers of the left ventricle (LV). Longitudinal shortening, circumferential shortening, radial thickening, LV myocardium rotation, and twist can be quantified using VVI [7–9]. Studies from the last decade have proven the usefulness of VVI for investigating various cardiac anomalies. For example, Chen et al. [7] used this method to examine myocardial systolic function in patients with hypertension and left ventricular hypertrophy, while Jarnert et al. [9] used VVI to examine the heart of diabetic patients.

In an initial pilot study [10], we used VVI to comprehensively assess the longitudinal strain (LS) of each LV segment and wall of the endocardium, myocardium, and epicardium of patients with CA; the endocardial LS during LV systole (LV ENDO LSsys) was the most sensitive and specific parameter for CA detection among the myocardial mechanical characteristics.

Therefore, the main objective of the present study was to identify independent factors that could influence the prognosis of Chinese patients with CA, among demographic characteristics, clinical manifestations, laboratory testing indicators, electrocardiographic features, conventional echocardiographic parameters, and LS determined using LV endocardial VVI. We also aimed to examine the survival of these patients.

## 2. Methods

### 2.1. Subjects

This was a prospective cohort study of 72 patients diagnosed with CA and admitted to the PLA General Hospital between November 2017 and April 2021. The exclusion criteria were as follows: (1) acute or previous myocardial infarction or (2) with moderate or severe aortic valve lesions. In the present study, all patients with hypertrophy and suspected CA underwent echocardiography and cardiac magnetic resonance imaging (MRI) examination to confirm the diagnosis. This study was approved by the ethics committee of the PLA General Hospital (approval ID: 2012051). All enrolled subjects provided a written informed consent.

CA was diagnosed on the basis of the following criteria [11]: (1) confirmed presence of systemic amyloid deposition on pathological examination of noncardiac tissues, (2) radiographic evidence of cardiac involvement (echocardiographic examination showing left ventricular hypertrophy and myocardial thickening ≥ 12 mm accompanied by a granular echo in the myocardium, and cardiac MRI showing the typical characteristics of delayed enhancement), and (3) the diagnosis of AL based on the evidence of plasma cell dyscrasia or by identification of an immunoglobulin light chain in their amyloid deposits or both.

### 2.2. Data Collection

**Figure 1:**
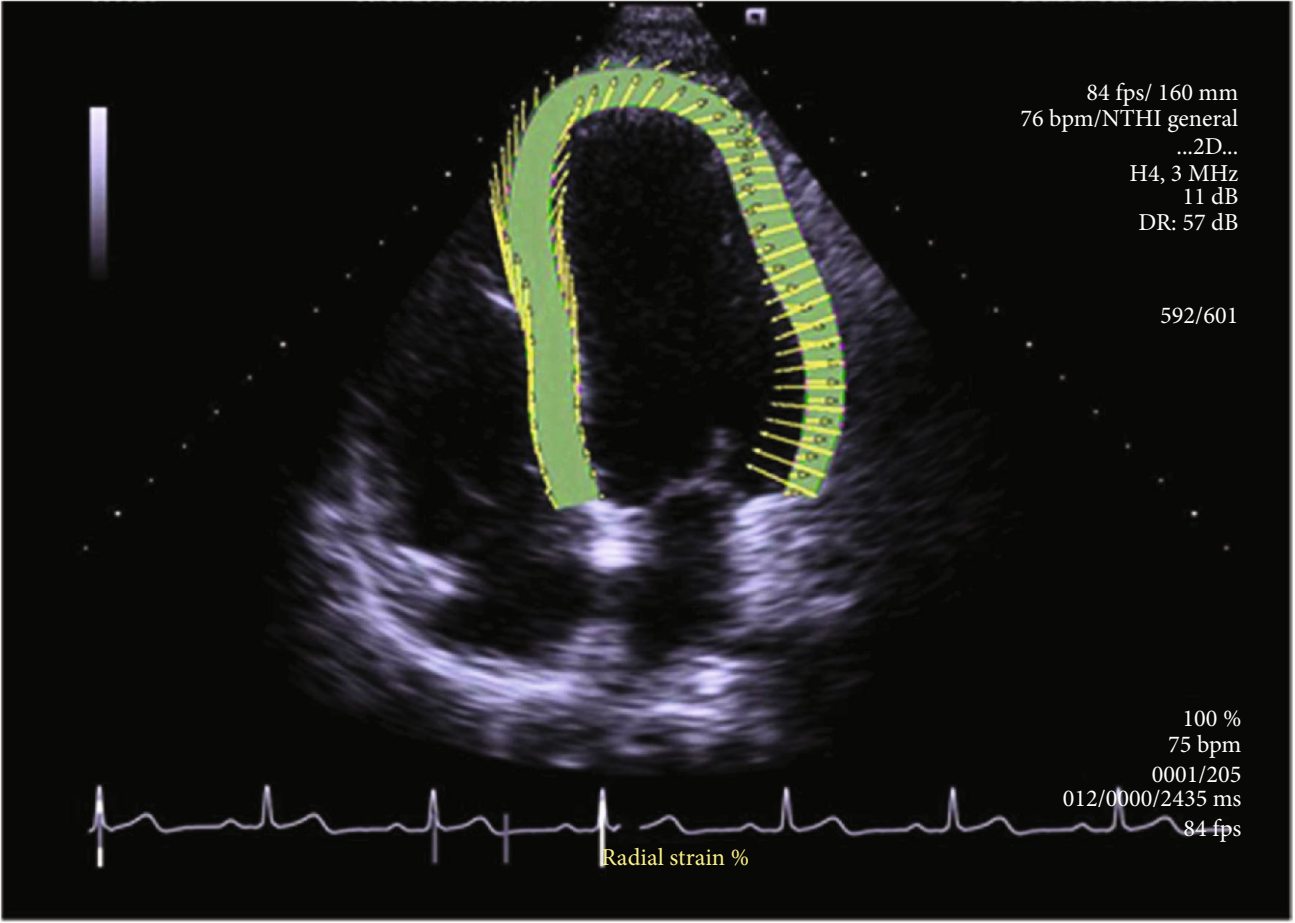
VVI was used to analyze the ventricular apical four-chamber section of the cardiac LS of the endocardium in a healthy individual. Only the left ventricular border was drawn in the corresponding section; the software could automatically generate the LS longitudinal motion direction and displacement and calculate the LS parameters.

The following data were recorded within 2 weeks after diagnosis: demographics, clinical symptoms, results of physical examination, and laboratory data (routine blood tests, i.e., complete blood count and hemoglobin (Hb) assay; blood chemistry, i.e., creatinine (Cr), proBNP, TnT, creatine kinase (CK), and creatine kinase isoenzyme (CK-MB) assays; and 24 h urinary proteins); results of ECG (including rhythm, conduction abnormalities, low voltage in the limb leads, pseudo-Q-wave infarction, and left ventricular hypertrophy), echocardiography (including thickness of the interventricular septum (IVS) and left ventricular posterior wall (LVPW), LV end-diastolic and end-systolic diameters (LVEDD and LVESD, respectively) and volume (LVEDV and LVESV, respectively), state of the left atrium, LV chamber size (LA and LV), LV ejection fraction (LVEF), ratio of mitral peak early diastolic flow velocity (E), mitral peak late diastolic blood flow velocity (A), E-peak deceleration time (DT), and pericardial effusion (PE)), chest radiography, and abdominal ultrasound or computed tomography; and results of bone marrow aspiration and noncardiac tissue biopsy examination. All blood cell analyses were performed using an automatic blood cell analyzer. All blood biochemistry analyses were carried out using an automated chemiluminescence system using all original reagents from the manufacturer. Urinary proteins were assessed using a BS-480 analyzer. For VVI imaging, clear apical four-chamber, two-chamber, and three-chamber views were selected for imaging. Two-dimensional dynamic grayscale images of three consecutive cardiac cycles were recorded and saved at 70–105 frames/second (Figure 1). Then, ENDO LSsys values for each LV segment and wall were automatically obtained from apical views using the analysis module of the software. ENDO LSsys values for basal, mid, and apical circular levels of LV were obtained by averaging the corresponding strain values of all segments at each level. Global LV ENDO LSsys was obtained by averaging the corresponding strain values of all 16 segments. All data were obtained as the average values of three consecutive cardiac cycles.

### 2.3. Follow-Up and Survival

The diagnosis was the starting point of observation. All-cause mortality was the endpoint. Follow-up was censored on September 30, 2021. Survival status was monitored by the same doctors every 3 months by phone. The patients received outpatient or hospital follow-up (including assessment of clinical symptoms and signs, routine blood tests, and echocardiography) every 6 months. The endpoint event (death) was determined by the local police or on the basis of a death certificate provided by a hospital. Patients who could not be reached three consecutive times were considered lost to follow-up.

### 2.4. ECG

Patients underwent standard 12-lead ECG in the supine position. ECG indicators for examination were rhythm, conduction abnormalities (e.g., atrioventricular block and right or left bundle branch block), low voltage in the limb leads (sum of Q-, R-, and S-wave voltage absolute values was equal to or less than 0.5 mV), pseudo-Q-wave infarction (pathological Q-waves were visible in ECG and were ≥2 contiguous or relevant leads, but coronary angiography or coronary CT revascularization showed normal findings), and left ventricular hypertrophy (the sum of V1-lead S waves and V5- or V6-lead R waves for females was ≥3.5 mV and that for males was ≥4.0 mV).

### 2.5. Cardiac Ultrasound

Ultrasonic diagnostic apparatus (ACUSON SC2000, Siemens, Erlangen, Germany) with a 4V1c probe was used to acquire two-dimensional echocardiographic images, Doppler images, and VVI images. The SC2000 eSie VVI software was used to process VVI images. The main observation indicators for conventional echocardiography were thickness of the interventricular septum (IVS) and left ventricular posterior wall (LVPW), LV end-diastolic and end-systolic diameters (LVEDD and LVESD, respectively) and volume (LVEDV and LVESV, respectively), state of the left atrium, LV chamber size (LA and LV), LV ejection fraction (LVEF), ratio of mitral peak early diastolic flow velocity (E), mitral peak late diastolic blood flow velocity (A), E-peak deceleration time (DT), and pericardial effusion (PE). Doppler imaging (TDI) was used to measure the maximum mitral annulus early myocardial diastolic velocity e′ and the E/e′ ratio.

For VVI imaging, patients diagnosed with CA lay on the examination table in the left lateral position, and coordinated ECG monitoring was conducted. Clear apical four-chamber, two-chamber, and three-chamber views were selected for imaging. Two-dimensional dynamic grayscale images of three consecutive cardiac cycles were recorded and saved at 70–105 frames/second. The SC2000 eSie VVI software was used for parameter analysis. First, the analysis interface of the software was accessed, the point where the endocardium of the left ventricle was clearly exposed was selected and free-framed in the three abovementioned views, and the ventricular endocardial border was draw manually. Next, the software automatically traced the epicardial boundary, with manual adjustments when necessary (Figure 1). Then, ENDO LSsys values for each LV segment and wall were automatically obtained from apical views using the analysis module of the software. ENDO LSsys values for basal, mid, and apical circular levels of LV were obtained by averaging the corresponding strain values of all segments at each level. Global LV ENDO LSsys was obtained by averaging the corresponding strain values of all 16 segments. All data were obtained as the average values of three consecutive cardiac cycles.

### 2.6. Statistical Analysis

Continuous data were presented as mean ± standard deviation, while categorical data were presented as frequencies. Survival was estimated using the Kaplan-Meier analysis. Survival comparison was performed using the log-rank test. Univariate and multivariate analyses were performed using the Cox proportional hazard regression model. Demographics, clinical features, imaging (including ENDO LSsys of regional parts (six walls and three circular levels) and global), and laboratory findings were analyzed using univariate methods. For the latter analysis, the model had three modes: clinical features alone, clinical features + laboratory findings, and clinical features + laboratory findings + echocardiographic data. All statistical analyses were performed using SPSS 21.0 (IBM, Armonk, NY, USA). Two-sided *P* values < 0.05 were considered statistically significant.

## 3. Results

### 3.1. Characteristics of the Patients

This study enrolled 72 patients diagnosed with CA. On the basis of mortality during follow-up, they were divided into the nonsurvival group (*n* = 39) and the survival group (*n* = 33).  Table 1 shows that there was no difference in gender, age, disease subtype, heart rate, systolic blood pressure, hemoglobin levels, CK-MB levels, Cr levels, 24 h urinary proteins, liver function, atrial fibrillation, and ECG between the two groups (all *P* > 0.05). But there were more patients with advanced NYHA class in the nonsurvival group (*P* < 0.001), as well as more patients with multiple involved organs (*P* = 0.02), higher log NT-proBNP levels (*P* = 0.002), and higher TnT levels (*P* = 0.01) (Table 1).

### 3.2. Echocardiography

Table 2 shows the comparison of the routine baseline echocardiographic data between the two groups. Left ventricular systolic function between the nonsurvival group and the survival group had no significant difference (all *P* > 0.05), but the thickness of the interventricular septum and the left ventricular posterior wall of the nonsurvival group was significantly increased compared to that of the survival group (all *P* < 0.05). Meanwhile, left diastolic function parameters of E/A, E/e′, were significantly increased in the nonsurvival group than the survival group (all *P* < 0.05). Table 2 also shows a comparison of ENDO LSsys for each wall and each circular level of LV and the global LV ENDO LSsys. A significant decrease was found in ENDO LSsys of the LV septum, anterior septum, posterior wall, basal and middle levels of LV, and overall LV for the nonsurvival group compared to the survival group (all *P* < 0.05).

### 3.3. Follow-Up and Survival

The longest follow-up was 47 months. The mean follow-up was 17.1 ± 12.9 months. Among the 72 patients, 39 died, 23 survived, and 10 were lost to follow-up. Thirty patients (70%) with AL-CA received systemic blood chemotherapy and biological treatments; of these, five patients underwent autologous bone marrow stem cell transplantation.

**Figure 2:**
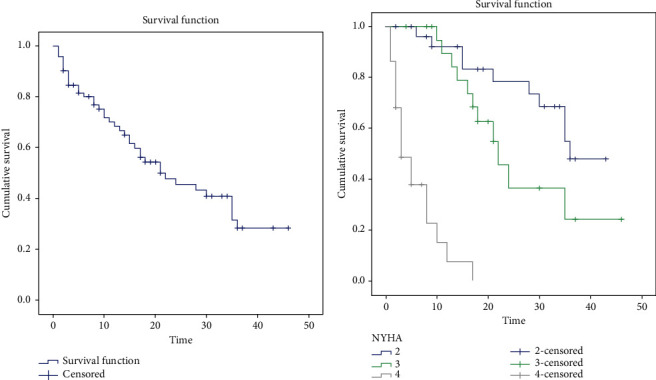
(a) Survival curve of all patients with CA. (b) Comparison of survival curves according to NYHA classes.

Mean survival for all patients was 24.7 ± 2.2 months, and the median survival was 22.0 months (Figure 2(a)). Mean survival was 32.7 ± 2.4 months among patients with NYHA class II, 26.6 ± 3.4 months for NYHA class III, and 5.8 ± 1.1 months for NYHA class IV (Figure 2(b)).

### 3.4. Identification of Independent Prognostic Factors for CA

Table 3 shows the univariate Cox regression analyses. NYHA class (HR = 3.99, 95% CI: 2.16–7.40, *P* < 0.001), log NT-proBNP level (HR = 3.32, 95% CI: 1.59–6.95, *P* = 0.001), TnT levels (HR = 1.72, 95% CI: 1.09–3.11, *P* = 0.02), thickness of the LVPW posterior wall (HR = 1.10, 95% CI: 1.01–1.17, *P* = 0.03), E/e′ (HR =1.10, 95% CI: 1.02–1.18, *P* = 0.01), and ENDO LSsys of LV IVS (HR = 1.10, 95% CI: 1.03–1.36, *P* = 0.01), LV POS (HR = 1.10, 95% CI: 1.08–1.49, *P* = 0.01), LV basal level (HR = 1.56, 95% CI: 1.08–1.47, *P* = 0.004), and global ENDO LSsys (HR = 1.11, 95% CI: 1.01–1.36, *P* = 0.01) were identified as factors that may have an impact on the prognosis of patients with CA.

The multivariate Cox proportional hazard regression model was subsequently used to identify independent prognostic factors for CA with three modes of analysis: clinical features alone, clinical features + laboratory findings, and clinical features + laboratory findings + echocardiographic data (Table 4). As seen in Table 4, NYHA class (HR = 3.42, 95% CI: 1.36–8.65, *P* = 0.002), log-proBNP level (HR = 1.40, 95% CI: 1.17–5.83, *P* = 0.03), and ENDO LSsys of the LV basal level (HR =1.25, 95% CI: 1.05–1.95, *P* = 0.004) were independent prognostic factors for CA.

## 4. Discussion

CA has a poor prognosis [4, 5, 11–13], but studies on the prognosis of Chinese patients with CA are limited. This study was aimed at identifying independent prognostic factors for CA in Chinese patients and at examining cardiac mechanical parameters using velocity vector imaging (VVI) as a potential prognostic factor. In this study, the mean follow-up was 17.1 ± 12.9 months, and mean survival for all patients was 24.7 ± 2.2 months. Mean survival decreased with increasing NYHA class. The multivariate Cox proportional hazard regression model showed that NYHA class, proBNP level, and ENDO LSsys of the LV basal level were independent prognostic factors for CA.

Systemic amyloidosis is a multisystem disease due to the deposition of amyloid substances in various organs, and its annual incidence is about 6–10 per 100 million people [14]. AL-CA, the most common subtype of CA, is caused by the deposition of monoclonal immunoglobulin light chain generated by bone marrow plasma cells and is associated with a poor prognosis, with reported median survival of 6–24 months [5, 15].

In the present study, the mean survival of the overall cohort was 24.7 ± 2.2 months, and the median survival was 22.0 months. The survival in the present study was longer than in previously published reports [5, 15]. This could be explained by two facts: (1) the cardiac function of our cohort was better than that of previous studies and (2) our patients received systemic medication and showed compliance after discharge from the hospital.

Since the prognosis of CA patients is generally poor, it is important to investigate the factors that affect the prognosis of CA in order to achieve early recognition of the disease progression. Several studies [5, 13, 16–18] have demonstrated the prognostic usefulness of cardiovascular functional assessment in patients with CA using the NYHA class, cardiac biomarkers, and conventional echocardiographic parameters such as LVEF, E/A, and E/e′. However, the prognosis of CA in Chinese patients and the factors that influence its prognosis are poorly known. Further, to our knowledge, no previous study applied advanced echocardiographic cardiac mechanical parameters such as LV ENDO LSsys for the analysis of CA prognosis.

The present study showed that ENDO LSsys at IVS, POS, and ASE walls and basal level and of global LV were associated with CA prognosis, suggesting that the ENDO LSsys, as a parameter of myocardial motion mechanics, not only reflects changes in deformation ability of the heart of CA patients but also plays a role in the prognosis of CA. The multivariate analysis showed that the NYHA class, proBNP level, and ENDO LSsys were independent factors that could predict the prognosis of CA. NYHA cardiac function class has been identified as an independent predictive factor of CA prognosis in several studies, both domestic and international [16, 19], and its impact on the prognosis of CA is more significant than that of many other clinical signs and symptoms. Irrespective of whether it was analyzed alone (model 1) or in combination with laboratory data and echocardiographic data (models 2 and 3), the NYHA class, as well as the proBNP level and LS of the endocardium at the LV basal level, was always identified as an independent predictive factor for the prognosis of CA. The NT-proBNP and TnT levels were also identified as factors predictive of CA prognosis in the univariate analysis. The reason why the TnT levels were not identified in the multivariate analysis may be that this parameter is related to other prognostic parameters such as the NYHA class, NT-proBNP level, and cardiac strain parameters, and these reflect the extent of myocardial damage better than TnT levels. Recently, LS determined by echocardiographic technologies has become an international hot topic for the diagnosis and evaluation of cardiac disease, but not many studies have examined LV ENDO LSsys in patients with CA. The present study showed that although both the ENDO LSsys of the global LV and ENDO LSsys of the basal level of LV were related to the prognosis of CA, the latter was an independent predictive factor for CA prognosis. These results are supported by pathology results; indeed, Hosch et al. [20] showed that amyloid was mainly invading the subendocardial myocardium. In addition, Brenner et al. [21] observed that the immunoglobulin light chain more easily damaged the myocardial basal part through oxidative stress. Therefore, the subendocardial and basilar parts of the myocardium seem to be involved earlier than other cardiac parts in heart damage of CA. Our previous studies also demonstrated that the LS of the endocardium in the left ventricular systole examined by VVI sensitively reflects myocardial mechanical damage in CA patients [10].

This study has some limitations. Although the number of observed indicators was quite high, the sample size of this study was small, and the follow-up was limited. In future studies, we will include a larger sample and increase the follow-up time, whereby we may be able to identify more accurate and reliable factors that could predict CA prognosis. In addition, we treated 10 patients who were lost to follow-up as living patients, which may affect the results of this study.

## 5. Conclusion

The present study strongly suggests that NYHA class, proBNP level, and ENDO LSsys of the basal level of LV were independently associated with survival of patients with CA. Therefore, these parameters can be used clinically to predict the prognosis of CA patients and may form the basis for an effective treatment method for these patients.

## Figures and Tables

**Table 1:** Baseline demographic and clinical data according to survival.

Characteristics	Nonsurvival group (*n* = 39)	Survival group (*n* = 33)	*P*
Gender (M/F)	31/8	26/7	0.82
Age (years)	56.0 ± 11.2	51.7 ± 12.5	0.43
Classification (*n*)			0.56
AL-CA	25	18	
Non-AL-CA	14	15	
NYHA class (*n*)			<0.001
II	3	20	
III	22	12	
IV	14	1	
Heart rate (bpm)	83.1 ± 11.3	78.9 ± 12.5	0.14
Systolic pressure (mmHg)	109.5 ± 10.4	113.3 ± 12.8	0.17
Other organs involved (*n*)			0.02
0	5	15	
1	17	11	
2	10	5	
3	7	2	
Hemoglobin (g/L)	110.8 ± 15.3	115.3 ± 11.2	0.17
Log NT-proBNP (pg/mL)	4.22 ± 1.7	2.76 ± 2.1	0.002
TnT (ng/mL)	0.14 ± 0.1	0.08 ± 0.1	0.013
CK-MB (ng/mL)	5.86 ± 4.7	4.71 ± 3.1	0.26
Cr (*μ*mol/L)	121.4 ± 32.2	109.7 ± 28.5	0.11
24 h urinary protein (mg/24 h)	1.48 ± 0.5	1.53 ± 0.6	0.70
ALT (U/L)	24.4 ± 16.2	29.8 ± 18.5	0.19
AST (U/L)	24.9 ± 11.9	30.0 ± 18.3	0.16
ALP (U/L)	130.6 ± 55.1	108.6 ± 60.7	0.11
Atrial fibrillation (*n*)	10	9	0.91
Electrocardiogram (*n*)			
Low left ventricular voltage	14	10	0.80
Poor R wave progression	9	10	0.67
Fake pathological Q-wave	10	8	0.89

NYHA: New York Heart Association; NT-proBNP: N-terminal precursor of brain natriuretic peptide; TnT: troponin T; CK-MB: creatinine kinase; Cr: creatinine; ALT: alanine aminotransferase transferase; AST: glutamate aminotransferase; ALP: alkaline phosphatase.

**Table 2:** Echocardiographic characteristics according to survival.

Parameters	Nonsurvival group (*n* = 39)	Survival group (*n* = 33)	*P*
LA diameter (mm)	49.3 ± 6.6	49.5 ± 9.6	0.91
RA diameter (mm)	44.5 ± 7.0	43.8 ± 7.2	0.68
LVESD (mm)	35.1 ± 7.0	32.2 ± 9.3	0.14
LVEDD (mm)	46.2 ± 7.8	45.1 ± 7.4	0.54
LVESV (mL)	53.0 ± 25.4	52.2 ± 21.1	0.89
LVEDV (mL)	97.9 ± 35.2	102.5 ± 31.4	0.56
LVEF (%)	47.7 ± 10.1	48.8 ± 12.6	0.49
Thickness of IVS (mm)	18.4 ± 6.6	15.8 ± 6.3	0.09
Thickness of LVPW (mm)	16.9 ± 5.0	13.6 ± 3.1	0.002
E velocity (m/s)	0.83 ± 0.2	0.77 ± 0.3	0.32
A velocity (m/s)	0.59 ± 0.3	0.52 ± 0.3	0.31
E/A	1.72 ± 0.7	1.39 ± 0.5	0.03
DT (ms)	167 ± 44	188 ± 49	0.06
e′ velocity (m/s)	0.045 ± 0.018	0.063 ± 0.028	0.01
E/e′	18.2 ± 7.5	13.8 ± 6.1	0.009
PE (*n*)	25	20	0.95
ENDO LSsys for LV walls (cm/s)			
IVS	−5.1 ± 3.1	−8.3 ± 5.5	0.003
LAT	−10.1 ± 3.6	−11.8 ± 6.1	0.15
INF	−11.3 ± 4.8	−12.9 ± 6.2	0.22
ANT	−10.7 ± 6.1	−12.1 ± 7.3	0.38
POS	−8.3 ± 4.4	−11.5 ± 6.5	0.02
ASE	−5.8 ± 3.9	−8.8 ± 6.2	0.01
ENDO LSsys for LV levels (cm/s)			
Basal	−6.0 ± 3.8	−11.2 ± 6.0	<0.001
Mid	−8.3 ± 5.3	−12.6 ± 7.6	0.01
Apical	−14.5 ± 6.2	−15.8 ± 7.3	0.42
ENDO LSsys for global LV	−11.4 ± 5.5	−14.8 ± 6.4	0.02

LA: left atrium; RA: right atrium; LVESD: left ventricular end-systolic diameter; LVEDD: left ventricular end-diastolic diameter; LVESV: left ventricular systolic volume; LVEDV: left ventricular end-diastolic volume; EF: ejection fraction; LVPW: left ventricular posterior wall; E: mitral early diastolic flow velocity; A: mitral flow velocity of late diastolic phase; DT: E-wave deceleration time; e′: early diastolic velocity at attachment position of mitral septal myocardial tissue; PE: pericardial effusion; ENDO LSsys for LV: endocardial longitudinal strain during left ventricle systole; IVS: interventricular septum; LAT: lateral wall; ANT: anterior wall; INF: inferior wall; POS: posterior wall; ASE: anterior septum.

**Table 3:** Univariate Cox regression analysis.

Factors	HR	95% CI	*P*
NYHA class	3.99	2.16–7.40	<0.001
Log NT-proBNP (pg/mL)	3.32	1.59–6.95	0.001
TnT (ng/mL)	1.72	1.09–3.11	0.02
Thickness of LVPW (mm)	1.10	1.01–1.17	0.027
E/e′	1.10	1.02–1.18	0.012
ENDO LSsys of LV IVS	1.10	1.03–1.36	0.010
ENDO LSsys of LV POS	1.10	1.08–1.49	0.010
ENDO LSsys of LV basal level	1.56	1.08–1.47	0.004
ENDO LSsys of the global LV	1.11	1.01–1.36	0.011

NYHA: New York Heart Association; NT-proBNP: N-terminal precursor of brain natriuretic peptide; LVPW: left ventricular posterior wall; TnT: troponin T; E: mitral early diastolic flow velocity; e′: early diastolic velocity at attachment position of mitral septal myocardial tissue; ENDO LSsys of LV: endocardial longitudinal strain during left ventricle systole; LV: left ventricle; IVS: interventricular septum; POS: posterior wall; ASE: anterior septum.

**Table 4:** Multivariate Cox proportional hazard regression.

Models	HR	95% CI	*P* value
Model 1: clinical features			
NYHA class	3.79	2.05–7.00	<0.001
Model 2: clinical features + laboratory test results			
NYHA class	3.81	1.74–8.37	0.001
Log NT-proBNP	1.87	1.06–4.77	0.036
Model 3: clinical features + laboratory test results + echocardiographic parameters			
NYHA class	3.42	1.36–8.65	0.002
Log NT-proBNP	1.40	1.17–5.83	0.03
ENDO LSsys of LV basal level	1.25	1.05–1.95	0.004

NYHA: New York Heart Association; NT-proBNP: N-terminal precursor of brain natriuretic peptide; ENDO LSsys of LV: endocardial longitudinal strain during left ventricle systole.

## Data Availability

The data used to support the findings of this study are available from the corresponding author upon request.
